# Gut microbiota-mediated metabolic restructuring aggravates emotional deficits after anesthesia/surgery in rats with preoperative stress

**DOI:** 10.3389/fimmu.2022.819289

**Published:** 2022-08-08

**Authors:** Lei Lei, Muhuo Ji, Jinjin Yang, Sai Chen, Hanwen Gu, Jian-jun Yang

**Affiliations:** ^1^ Department of Anesthesiology, Pain and Perioperative Medicine, The First Affiliated Hospital of Zhengzhou University, Zhengzhou, China; ^2^ Henan Province International Joint Laboratory of Pain, Cognition, and Emotion, Zhengzhou, China; ^3^ Department of Anesthesiology, The Second Affiliated Hospital, Nanjing Medical University, Nanjing, China

**Keywords:** gut microbiota, microbial metabolites, postoperative emotional deficits, preoperative stress, neuroinflammation

## Abstract

Patients with preoperative stress are prone to postoperative emotional deficits. However, the underlying mechanisms are largely unknown. Here, we characterize the changes of microbial composition and specific metabolites after anesthesia/surgery in rats with preoperative stress based on 16S rRNA gene sequencing and non-targeted metabolomics technique. Consequently, we found that anesthesia/surgery aggravated anxiety-like and depression-like behaviors in rats under preoperative stress. Microglia were activated and pro-inflammatory cytokines, including interleukin 6 (IL-6) and tumor necrosis factor ɑ (TNF-α) were upregulated after anesthesia/surgery. The postoperative gut microbiota and metabolite composition of rats exposed to preoperative stress differed from those of control rats. Lastly, emotional impairments, metabolic alterations, and neuroinflammation returned normal in antibiotics-treated rats. Our findings provide further evidence that abnormalities in the gut microbiota contribute to postoperative metabolic restructuring, neuroinflammation, and psychiatric deficits in rats under preoperative stress.

## Introduction

Postoperative mental condition for patients is significantly correlated with physical outcomes and can dramatically influence the quality of life, thus leading to public health concerns (1, 2). Individuals with preoperative stress are prone to postoperative emotional deficits, especially adolescent patients (3). So considerable efforts are needed to reduce the risks for exacerbations or relapses of imminent psychiatric illness in the postoperative period in patients with high psychological vulnerability (4). However, the mechanisms for postoperative emotional deficits are largely unknown.

Preoperative stress arises from the combination of genetic, environmental, and experiential factors, with the latter two factors having the greatest impacts on sensitive development periods (5, 6). Specifically, recent robust discoveries from diverse disciplines, encompassing human studies and experimental models suggested that early-life adversity influences the maturation of brain circuits involved in cognition, especially in the perinatal period (7–10). Here, it was focused on the subjects with poor early life experiences that are the basis of preoperative stress.

Clinic data on postoperative psychiatric health offer promising outcomes, while the internal mechanisms are not clear (11). The evidence of gut microbiota perturbations has been accumulated for multiple neurodegenerative diseases, including postoperative cognitive decline (12–14). It was attempted to determine the causal linkage between gut microbiota and postoperative emotional deficits. Moreover, the underlying driving force that could promote gut microbiota-induced disturbances in specific brain areas remains obscure. Growing evidence has indicated that certain microbial members possess the capacity to synthesize and regulate neurochemicals to control neurotransmission and other metabolic molecules, which indirectly or directly affect neuronal activities (15, 16). Therefore, the identification of the specific metabolites associated with psychiatric disorders after anesthesia/surgery in rats under preoperative stress is of great importance. The current manuscript initially establishes a stress model, and individuals exposed to prenatal stress have susceptibility tendency. Stresses later in life such as anesthesia/surgery are triggers that allow the vulnerability to emerge as psychosis.

## Materials and methods

### Animals and antibiotics-treatment

All animal procedures were performed following the National Institute of Health Guidelines and with approval of the Animal Care and Use Committee of The First Affiliated Hospital of Zhengzhou University, Zhengzhou, China. Efforts were made to minimize the number of animals used. Adult Sprague-Dawley rats were bred under controlled illumination (12-h light and dark cycle, lights on at 7 a.m.), temperature (20-22°C), and relative humidity (50-60%) in polysulfone cages (375×270×180 mm) with free access to food and water. Cages (including litter) were changed twice a week and lids were changed once a week. Used cages were cleaned with disinfectant and autoclaved for later use. Female rats (70-80 days old) were mated with males (90-100 days old) at a ratio of 2:1 for 1 night and vaginal smears were examined by 6:00-8:00 a.m. the next day. The presence of a plug was considered a sign of successful mating, and continued mating was performed if no plug was found. The day after mating was recorded as the gestational day (G) 1 and pregnant rats were individually housed in the same environment until G11. Restraint stress was applied to pregnant rats from G12 to G18 to build a preoperative stress model and anesthesia/surgery stress was performed on the postnatal day (P) 35. Depending on the stress treatment, rats were randomly assigned to one of four groups: (1) CC group (no stress); (2) SC group (only preoperative stress); (3) CS group (only anesthesia/surgery stress); (4) SS group (preoperative stress and anesthesia/surgery stress). After the restraint stress, pregnant rats were left undisturbed until normal delivery. Litter sizes at birth ranged from 6 to 17 pups, and we culled litters to 6 pups on P2. To exclude the influence of estrogen, we only used male rats in our study. The control dams raised all pups until P21. At P22, pups were distributed (n=2/cage) according to their groups and were used for the following experiments. To mechanistically identify the correlation between the behavioral disturbance and gut microbiota, the effects of treatment with antibiotics were evaluated. SS rats were provided with sterile drinking water, supplemented with a cocktail of broad-spectrum antibiotics as previously described: ampicillin (0.1mg/ml), streptomycin (0.5mg/ml), and colistin (0.1mg/ml) (Sigma-Aldrich) (17). Antibiotic treatment was started from P28 and continued for seven days.

### Preoperative stress

Pregnant rats were subjected to chronic restraint stress (CRS) from G12 to G18 for 3 sessions per day (45 min per session starting at 09:00, 11:00, and 13:00 h) to build a preoperative stress model, during which they were placed in transparent plastic cylinders (inner diameter, 9 cm; length, 19 cm) and exposed to bright light (18). Rats from the control group were left undisturbed in their home cage.

### Anesthesia/surgery stress

Male rats from CS and SS groups were subjected to laparotomy under anesthesia as we described previously (19). Specifically, the P35 male rats were anesthetized in a temperature-controlled chamber (37°C) with 1.5% isoflurane in 100% oxygen (1.5 L/min). Thirty minutes after the induction, each rat was moved out of the chamber and an exploratory laparotomy was performed under aseptic conditions with 1.5% isoflurane: after shaving and sterilizing thoroughly with 0.5% iodine, and abdominal median incision of about 1 cm was made, to access the peritoneal cavity. The operator then moved the viscera, including the intestine and musculature. Absorbable 5-0 sutures were used to suture the incision layer by layer. EMLA cream (2.5% lidocaine and 2.5% prilocaine) was applied to the wound at the end of the procedure to prevent infection and pain. The surgical procedure for each rat lasted approximately 10 minutes, and then the rat was placed back into the anesthesia chamber to continue receiving anesthesia with 1.5% isoflurane for up to 2 h. Rats from the CC and SC group received 100% oxygen for 2 h.

### Behavioral tests

A well-trained investigator blinded to animal grouping performed the behavioral tests including the elevated plus maze (EPM at P43), the sucrose preference test (SPT at P44), and the forced swim test (FST at P47) as we described previously (20). Behavioral testing was carried out in the same order for all rats during the light phase of the circadian cycle (09:30–14:00).

### Elevated plus maze

The EPM consisted of 2 opposing open arms (50×10×0.5 cm) and 2 perpendicular opposing arms (50×10×45 cm) elevated 75 cm above the floor. Testing was carried out in a dimly lit room with a 40 W bulb hung 60 cm above the central part of the maze. Rats were placed in the center square facing an open arm and were allowed to explore the maze for 5 min. During the testing period, the rat’s behavior was recorded using Panlab SMART 3.0 video tracking software (RWD, Shenzhen, China). Animals who fell were removed from the study.

### Sucrose preference test

Beginning at P44, rats were habituated to drinking a solution of 1% sucrose for 24 h. At P45, rats were given the choice between a bottle containing normal drinking water and another containing sucrose solution. The bottles were left for 24 h, and we switched their positions after the first 12 h. At P46, we gave each rat 2 pre-quantified bottles, one containing normal drinking water and the other containing the sucrose solution. The test lasted 24 h, with the positions of the 2 bottles switched after the first 12 h. After 24 h, the bottles were removed and weighed. We calculated sucrose preference as the percentage of sucrose intake relative to the total fluid intake (water + sucrose).

### Forced swim test

Rats were placed in a plastic cylinder (diameter, 50 cm; height, 80 cm) filled with water (22°C to 25°C) for 6 min, and we recorded the floating time during the final 5 min. The water was changed between tests so that fresh water was used for each animal. We defined immobility as the absence of movement (leg kicks to stay afloat were not considered movement) as a measure of behavioral despair and helplessness (depression-like behavior). After the test, rats were removed from the water, dried with towels, and euthanized the next day under isoflurane anesthesia.

### Western blot

Pro-inflammatory cytokines interleukin 1β (IL-1β), IL-6, and TNF-ɑ of the hippocampus were evaluated using western blot. We extracted and quantified the total protein of hippocampal tissue. Equal protein amounts were loaded and separated by sodium dodecyl sulfate-polyacrylamide gel electrophoresis and transferred to a nitrocellulose membrane (Millipore, Billerica, MA, USA). Membranes were then blocked in 3% nonfat milk for 1 h at room temperature followed by overnight incubation at 4°C with primary antibodies against IL-1β (ABclonal, Woburn, MA, USA; A1112, 1:1000), IL-6 (ABclonal, Woburn, MA, USA; A0286, 1:1000), TNF-ɑ (ABclonal, Woburn, MA, USA; A11534, 1:1000), and β-actin (Proteintech, Chicago, IL, USA; 66009-1, 1:2000). Next, we incubated the membranes with appropriate secondary antibodies conjugated with horseradish peroxidase (1:10,000; Abbkine, Beijing, China) for 2 h at room temperature. Protein bands were detected using the ChemiDoc MP system (Bio-Rad, Hercules, CA, USA).

### Immunofluorescence

Brain sections were prepared and stained for IBA1 as previously described (18). All steps were carried out at room temperature unless otherwise specified. One day after the FST, we deeply anesthetized the rats with intraperitoneal phenobarbital sodium (60 mg/kg) followed by PBS and 4% paraformaldehyde. Brains were harvested, fixed in 4% paraformaldehyde overnight, and then dehydrated in 30% sucrose at 4°C. Samples were embedded in the Optimal Cutting Temperature compound and coronal sections (25 μm) were cut using a cryostat (Leica, Nussloch, Germany). Free-floating serial sections were washed in PBS and then serially incubated in PBS with 0.3% Triton for 10 min, in blocking buffer (10% normal goat serum and 0.1% Triton X-100 in PBS) for 60 min, and with rabbit anti-ionized calcium-binding adapter molecule 1 (IBA1, 1:1000; WAKO, 019-19741) overnight at 4°C. Sections were then washed in PBS and incubated for 2 h with Alexa Fluor 555-labeled goat anti-rabbit antibody (Jackson Immunoresearch, 111-165-045) diluted 1:500 in blocking buffer. Sections were washed again and mounted onto chromalum/gelatin-coated slides in the dark. For microglia images, we used confocal microscopy (Nikon, 1901680S) using 40× and 100× lenses. Image stacks were 5-μm thick with a z-step size of 0.5 μm, we analyzed them using the ImageJ software version 2.0.0 (NIH, Bethesda, MD).

### 16S rRNA gene sequencing and analysis

Total genome DNA from fecal was extracted using a CTAB/SDS method. We determined DNA concentration and assessed purity on 1% agarose gels. We diluted DNA samples to 1 ng/μL using sterile water. 16S rRNA/18SrRNA/ITS genes of distinct regions (16SV4/16SV3/16SV3-V4/16SV4-V5, 18S V4/18S V9, ITS1/ITS2, Arc V4) were amplified using specific primer pairs (*eg*, 16S V4: 515F-806R, 18S V4: 528F-706R, 18S V9: 1380F-1510R) with barcodes. All PCR reactions were carried out with the Phusion^®^ High-Fidelity PCR Master Mix (New England Biolabs).

We generated sequencing libraries using a TruSeq^®^ DNA PCR-Free Sample Preparation Kit (Illumina, USA) following the manufacturer’s recommendations and added index codes. The library quality was assessed on a Qubit@ 2.0 Fluorometer (Thermo Scientific) and an Agilent Bioanalyzer 2100 system. At last, the library was sequenced on an IlluminaHiSeq2500 platform generating 250-bp paired-end reads. To study phylogenetic associations of different operational taxonomic units (OTUs) and the difference of the dominant species in different samples (groups), we conducted multiple sequence alignments using the MUSCLE software (version 3.8.31). OTUs abundance data were normalized using a standard sequence number corresponding to the sample with the least sequences. Subsequent analysis of alpha and beta diversity was all performed regarding this output normalized data. We applied alpha diversity to analyze the complexity of species diversity for a sample through 6 indices, including Observed-species, Chao1, Shannon, Simpson, ACE, and Good-coverage. All these indices in our samples were calculated using the QIIME (version 1.7.0) software and displayed with R software (version 2.15.3). We applied beta diversity analyses to evaluate differences of samples in species complexity, beta diversity on both weighted and unweighted unifrac were calculated using the QIIME software (version 1.7.0). Cluster analysis was preceded by a principal component analysis (PCA) applied to reduce the dimension of the original variables using the FactoMineR package and ggplot2 package in the R software (version 2.15.3). We performed an unweighted pair-group method with arithmetic means (UPGMA) Clustering as a type of hierarchical clustering method to interpret the distance matrix using average linkage using the QIIME software (version 1.7.0).

### LC-MS/MS

To measure hydrophilic compounds, we thawed fecal on ice and then homogenized 50 mg with 500 µL of ice-cold methanol/water (70%, v/v). Next, samples were vortexed for 3 min, sonicated for 10 min in an ice water bath, and vortexed again for 1 min. Then, we centrifuged the samples at 12,000 rpm (4°C for 10 min). The collected supernatants were used for LC-MS/MS analysis. LIT and triple quadrupole (QQQ) scans were acquired on a triple quadrupole-linear ion trap mass spectrometer (QTRAP), QTRAP^®^ LC-MS/MS System equipped with an ESI Turbo Ion-Spray interface, operating in positive and negative ion modes and controlled using the Analyst 1.6.3 software (Sciex). The ESI source operation parameters were as follows: source temperature, 500°C; ion spray voltage (IS), 5500 V (positive), -4500 V (negative); ion source gas I (GSI) at 55 psi, gas II (GSII) at 60 psi, and curtain gas (CUR) at 25.0 psi; the collision gas (CAD) was high. Instrument tuning and mass calibration were performed with 10 and 100 μmol/L polypropylene glycol solutions in QQQ and LIT modes, respectively. A specific set of MRM transitions were monitored for each period according to the metabolites eluted within this period.

To measure hydrophilic compounds, we thawed samples on ice, took out 50 mg, and homogenized them with a 1-mL mixture (including methanol MTBE and the internal standard mixture) using a steel ball. After that, we took out the steel ball and shook the mixture for 2 min. We added 500 µL of water and shook the mixture for 1 min, and then centrifuged it at 12,000 rpm (4°C for 10 min). We concentrated 500 µL of supernatant. The powder was dissolved in 100 µL of mobile phase B, then stored at -80°C. Finally, we added the dissolving solution into the sample bottle for LC-MS/MS analysis. LIT and triple quadrupole (QQQ) scans were acquired on a triple quadrupole-linear ion trap mass spectrometer (QTRAP), QTRAP^®^ LC-MS/MS System, equipped with an ESI Turbo Ion-Spray interface, operating in positive and negative ion modes and controlled using the Analyst 1.6.3 software (Sciex). The ESI source operation parameters were as follows: ion source, turbo spray; source temperature, 550°C; ion spray voltage (IS), 5500 V; ion source gas I (GSI) set at 55 psi, gas II (GSII) set at 60 psi, and curtain gas (CUR) set at 25 psi; the collision gas (CAD) was medium. Instrument tuning and mass calibration were performed with 10 and 100 μmol/L polypropylene glycol solutions in QQQ and LIT modes, respectively. QQQ scans were acquired as MRM experiments with the collision gas (nitrogen) set at 5 psi. DP and CE for individual MRM transitions were done with further DP and CE optimization. A specific set of MRM transitions were monitored for each period according to the metabolites eluted within this period.

### Statistical analysis

We performed statistical analyses with the Prism v7.0 software (GraphPad, San Diego, CA, USA). Data are presented as means ± SEM. For comparison of two groups, we used unpaired *t*-tests for normally distributed data or Mann–Whitney U tests for non-normally distributed data. For comparisons between multiple groups, we applied two-way analysis of variance (ANOVA) followed by Tukey’s multiple comparisons test. Bivariate associations were evaluated using Pearson correlation coefficients. We considered all *P<*0.05 as statistically significant.

## Results

### Anesthesia/surgery aggravated behavioral deficits and neuroinflammation in rats with preoperative stress

**Figure 1:**
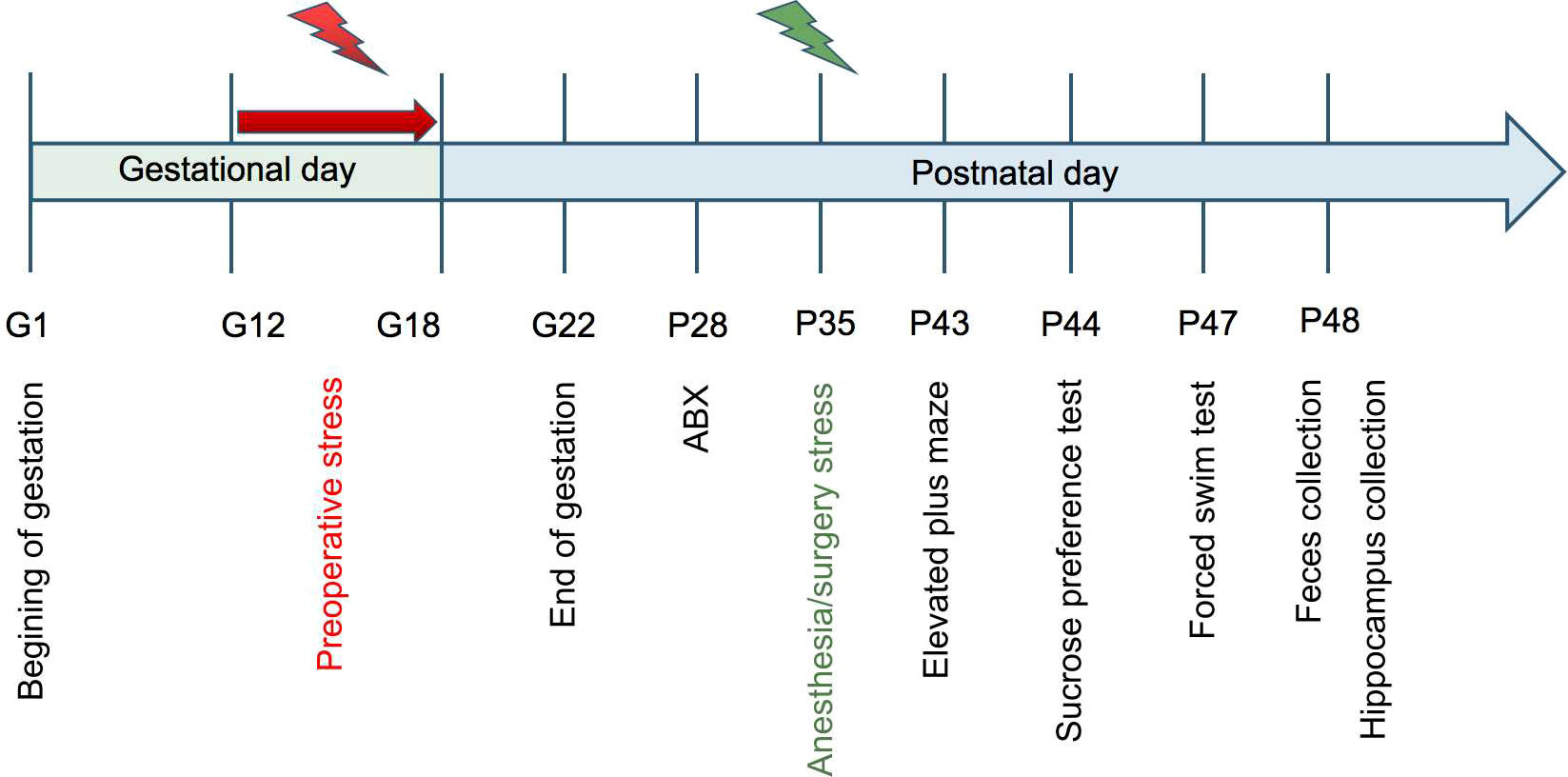
Experimental design Restraint stress was employed from G12 to G18 and ABX or vehicle was given for 7 consecutive days followed by anesthesia/surgery exposure on P35. A battery of behavioral tests was performed 7 days after anesthesia/surgery at P43-47 and fresh fecal and hippocampus samples were collected on the second day.

**Figure 2:**
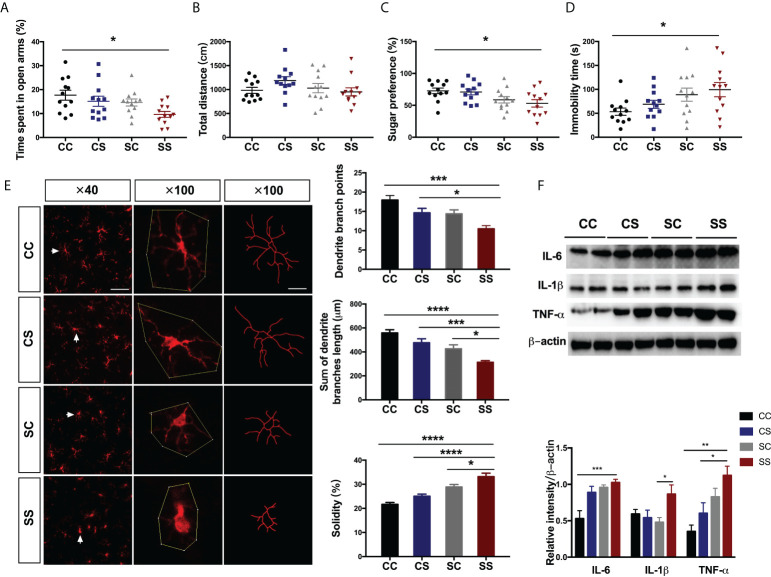
Anesthesia/surgery aggravated behavioral deficits and neuroinflammation in rats with preoperative stress **(A, B)** Time spent in the open arm **(A)** (n = 2/group) and total travel distance **(B)** (n = 12) in the four groups. **(C, D)** Sugar preference in SPT **(C)** (n = 12/group) and immobility time in FST **(D)** (n = 12/group) in the four groups. **(E)** Immunofluorescence staining and morphological analysis of microglia in the hippocampus (n = 16/group). Arrows denote the area represented in the inset. Scale bar = 50 µm; 20 µm for the inset. **(F)** Representative blot and quantitative analysis of IL-6, IL-1β, and TNF-α in the hippocampus (n = 6/group). One-way ANOVA and Tukey’s multiple comparisons test were used to analyze the data. Data represent mean ± SEM. *P<0.05, **P<0.01, ***P<0.001, ****P<0.0001 vs SS group. CC, no stress; SC, only preoperative stress; CS, only anesthesia/surgery stress; SS, preoperative stress and anesthesia/surgery stress.

To determine the effects of anesthesia/surgery on anxiety-like and depression-like behaviors in rats exposed to preoperative stress, we performed a classical behavioral paradigm in adolescent rats (**Figure 1**). The SS rats showed significantly less time in the open arm compared with the CC rats (preoperative stress: F[1, 44]=5.819, *P=*0.020; anesthesia/surgery stress: F[1, 44]=4.522, *P=*0.039, **Figure 2A**), but there was no significant difference between other groups. The total distances were not changed by anesthesia/surgery (**Figure 2B**) indicating that anesthesia/surgery could induce anxiety-like behaviors. Next, we performed SPT and FST to assess the depression-like behaviors and found that anesthesia/surgery caused significant changes in sugar preference (preoperative stress: F[1, 44]=10.22, *P=*0.003; anesthesia/surgery stress: F[1, 44]=0.534, *P=*0.469, **Figure 2C**) and immobility time (preoperative stress: F[1, 44]=8.179, *P=*0.007; anesthesia/surgery stress: F[1, 44]=1.258, *P=*0.268, **Figure 2D**).

Increasing evidence has demonstrated the interactions between neuroinflammation and postoperative cognitive disorders (19, 21). We asked whether microglia activation plays a role in the emotional deficits following anesthesia/surgery in rats exposed to preoperative stress. Notably, we found significant morphological differences of hippocampal microglia between SS and other groups. Specifically, the microglia of CC rats had small, thin, and highly bifurcated cell bodies consistent with the morphology of resting microglia. In contrast, cells in SS rats contained large cell bodies and thick branches, a morphology characteristic of activated microglia. We also quantified other parameters characterizing the cell morphology of microglia. SS rats showed shorter total dendritic branch lengths (preoperative stress: F[1, 60]=29.27, *P<*0.001; anesthesia/surgery stress: F[1, 60]=12.63, *P<*0.001, *P<*0.0001 vs CC group, *P<*0.001 vs CS group, *P=*0.026 vs SC group) and lower dendritic branch points (preoperative stress: F[1, 60]=12.96, *P<*0.001; anesthesia/surgery stress: F[1, 60]=11.33, *P=*0.001, *P<*0.0001 vs CC group, *P=*0.040 vs CS group) than those of other groups (**Figure 2E**). We calculated solidity as the ratio between the cell area and the convex area related to the spatial complexity of the cell. The rats in the SS group exhibited higher solidity than those of other groups (preoperative stress: F[1, 60]=52.86, *P<*0.001; anesthesia/surgery stress: F[1, 60]=13.32, *P<*0.001, *P<*0.0001 vs CC group, *P<*0.0001 vs CS group, *P=*0.026 vs SC group, **Figure 2E**). Consistent with the increased microglia activation after AS, we observed similar changes of pro-inflammatory cytokines IL-6 (preoperative stress: F[1, 20]=14.56, *P=*0.001; anesthesia/surgery stress: F[1, 44]=8.38, *P=*0.009, *P<*0.001 vs CC group) and TNF-ɑ (preoperative stress: F[1, 20]=17.04, *P<*0.001; anesthesia/surgery stress: F[1, 20]=5.09, *P=*0.035, *P=*0.001 vs CC group, *P=*0.030 vs CS group), but not IL-1β, in the hippocampus of SS rats (**Figure 2F**).

### Anesthesia/surgery induced changes in gut microbiota in rats with preoperative stress

**Figure 3:**
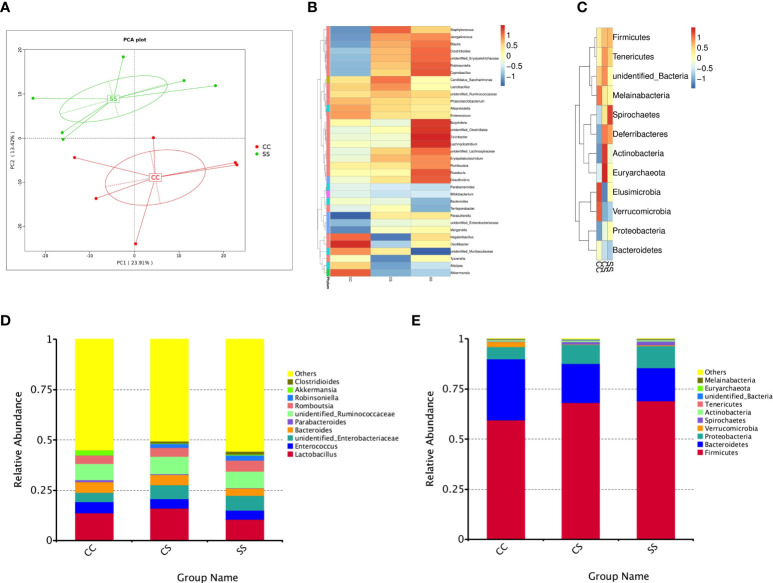
Anesthesia/surgery induced changes in gut microbiota in rats with preoperative stress **(A)** OUTs-based principal component analysis (PCA) from CC and SS group (n = 6/group). **(B, C)**Heatmap of bacterial communities at the genus level **(B)** and phylum level **(C)** for rats in the three groups (n = 6/group). **(D, E)** Top 10 species with the largest relative abundance at the genus level **(D)** and phylum level **(E)** for rats in the three groups (n = 6/group). CC, no stress; CS, only anesthesia/ surgery stress; SS, preoperative stress and anesthesia/surgery stress.

Given that gut microbiota could regulate the immune system and contributes to neuroinflammation, we investigated whether microbiota dysbiosis was associated with host emotional deficits after anesthesia/surgery in rats with preoperative stress (22, 23). The principal component analysis (PCA) revealed a remarkable difference in the gut microbiota composition between CC rats and SS rats (**Figure 3A**). Notably, taxonomic profiling demonstrated that the bacterial community was altered after anesthesia/surgery exposure (**Figure 3B** **–** **E**). 16S rDNA sequencing revealed the composition of gut microbiota changes due to the anesthesia/surgery in rats with preoperative stress both at the genus (**Figure 3B**) and phylum (**Figure 3C**) levels. Using OTUs to track the abundance of different bacterial taxa, we found significant changes in gut microbiota profiles in SS rats compared with other rats (**Figure 3D**). We also observed similar changes at phylum (**Figure 3E**) level. These data demonstrated that anesthesia/surgery exposure facilitates microbiota dysbiosis in rats exposed to preoperative stress.

### Anesthesia/surgery induced changes in metabolites in rats with preoperative stress

**Figure 4:**
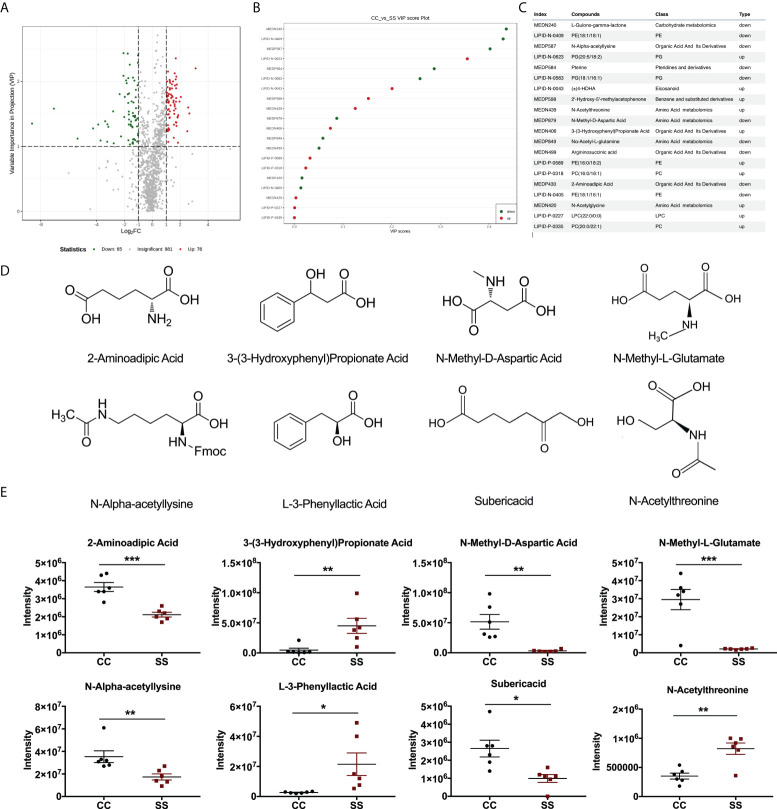
Anesthesia/surgery induced changes in metabolites in rats with preoperative stress **(A)** Volcano plot of differential metabolites in fecal samples from CC and SS rat group (n = 6/group). **(B)** Variable importance in projection (VIP) score plot of differential metabolites in fecal samples from CC and SS rat group (n = 6/group). **(C)** Part of the index numbers and corresponding metabolite compounds were detected in this experiment. **(D)** Structures of 2-aminoadipic acid, 3-(3-sulfooxyphenyl)propanoic acid, N-methyl-D-aspartic acid, N-methyl-L-glutamate, N-alpha-acetyl-lysine, L-3-Phenyllactic acid, suberic acid, and N-acetylthreonine. **(E)** Relative abundances of 2-aminoadipic acid, 3-(3-sulfooxyphenyl)propanoic acid, N-methyl-D-aspartic acid, N-methyl-L-glutamate, N-alpha-acetyl-lysine, L-3-Phenyllactic acid, suberic acid, and N-acetylthreonine in fecal samples from CC and SS rat group (n = 6/group). An unpaired t-test was used for normally distributed data and the Mann-Whitney U test was used for non-normally distributed data. Data represent mean ± SEM. *P<0.05, **P<0.01, ***P<0.001 vs SS group. CC, no stress; SS, preoperative stress and anesthesia/surgery stress.

The bacterial community metabolizes and synthesizes several metabolites that indirectly or directly affect the neuronal activities of the host (15, 16). We employed a non-targeted metabolomics technique to characterize the fecal metabolome using Ultra Performance Liquid Chromatography (UPLC) and Tandem mass spectrometry (MS). Among those metabolites matched to the database, we found that the abundance of 76 and 65 metabolites upregulated and downregulated respectively in SS rats as annotated by MWDB (metware database) (**Figure 4A**). Specially, we found 20 metabolites with significant changes (VIP≥1, *P<*0.05) *via* a multivariate statistical analysis (**Figure 4B**). These metabolic markers were mainly involved in the metabolism of amino acids, organic acids, and their derivatives. We identified six metabolites: 2-aminoadipic acid (Welch-corrected t[7.573]=5.486, *P<*0.001), 3-(3-sulfooxyphenyl) propanoic acid (Mann-Whitney U=1, *P=*0.004), N-methyl-D-aspartic acid (t[10]=3.96, *P=*0.003), N-methyl-L-glutamate (t[10]=4.90, *P<*0.001), N-alpha-acetyllysine (Mann-Whitney U=0.5, *P=*0.004), and suberic acid (Welch-corrected t[7.089]=3.228, *P=*0.014) that were significantly decreased in fecal samples of SS rats compared with those of CC rats. Two other metabolites, L-3-phenyllactic acid (t[10]=2.51, *P=*0.031) and N-acetylthreonine (Welch-corrected t[7.594]=4.264, *P=*0.003) were significantly more abundant in SS rats than those of CC rats (**Figure 4E**).

**Figure 5:**
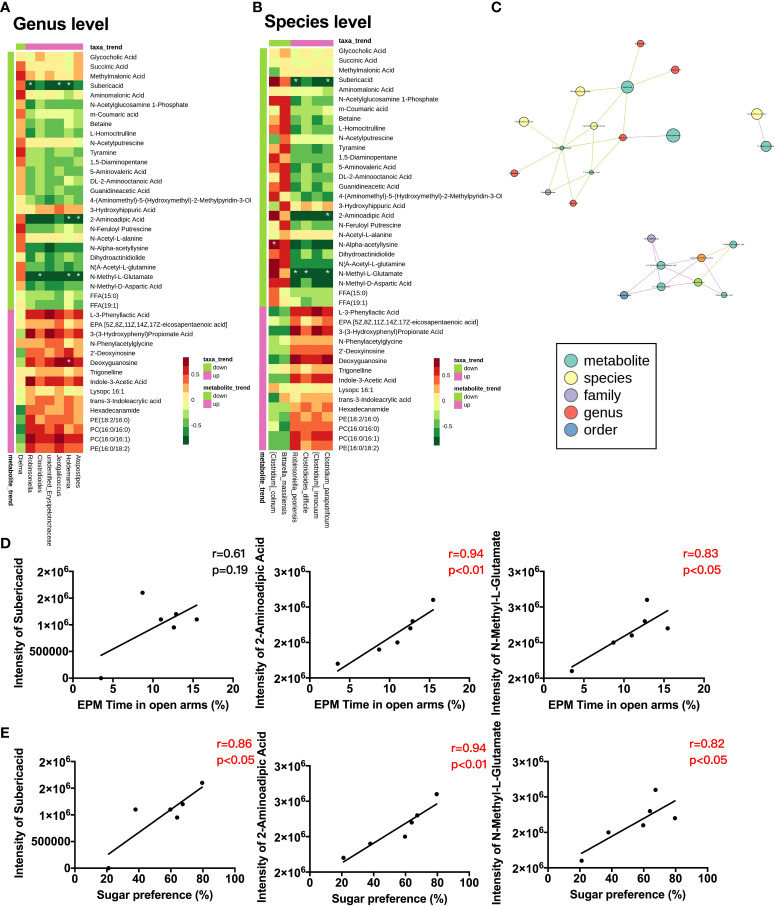
Correlations between host metabolites, gut microbiota, and behavioral changes **(A)** Correlation heatmap between metabolites and gut microbiota at genus level in CC and SS group (n = 6/group). **(B)** Correlation heatmap between metabolites and gut microbiota at the species level in CC and SS group (n = 4-6/group). **(C)** Total correlation network between metabolites and gut microbiota in CC and SS group (n = 4-6/group). **(D)** Correlations between metabolites and time spent in open arm in CC and SS group (n = 6/group). **(E)** Correlations between metabolites and sugar preference in CC and SS group (n = 6/group). *P<0.05. CC, no stress; SS, preoperative stress and anesthesia/surgery stress.

Next, we analyzed correlations between the metabolites and microbiota by calculating Spearman’s correlation coefficient to comprehensively evaluate their associations. The heatmaps showed a significant correlation between metabolites and microbiota at the genus (**Figure 5A**) and species (**Figure 5B**) levels. A network plot exhibits the total correlation between metabolites and gut microbiota (**Figure 5C**). We further assessed the possible predictive value of these metabolites on behavioral changes. Our results revealed that 2-aminoadipic acid and N-methyl-L-glutamate were positively correlated with the time spent in the open arm (**Figure 5D**) and the sugar preference (**Figure 5E**).

### Treatment with antibiotics prevented postoperative behavioral and metabolic changes in rats with preoperative stress

**Figure 6:**
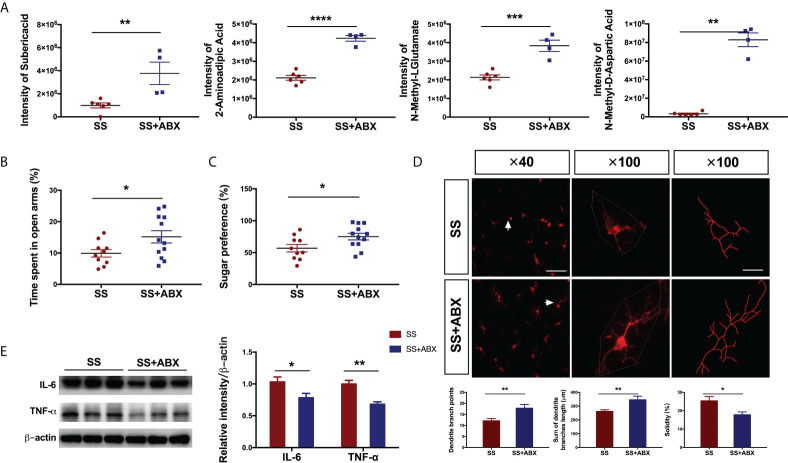
Antibiotics attenuated the anesthesia/surgery induced changes in rats with preoperative stress **(A)** Relative abundances of suberic acid, 2-aminoadipic acid, N-methyl-L-glutamate, and N-methyl-D-aspartic acid in fecal samples of SS and SS+ABX group (n = 4-6/group). **(B)** Time spent in the open arm of SS and SS+ABX group (n = 12/group). **(C)** Sugar preference in SPT of SS and SS+ABX group (n = 12/group). **(D)** Immunofluorescence staining and morphological analysis of microglia in the hippocampus of SS and SS+ABX group (n = 16/group). Arrows denote the area represented in the inset. Scale bar = 50 µm; 20 µm for inset. **(E)** Representative blot and quantitative analysis of IL-6 and TNF-α in the hippocampus of SS and SS+ABX group (n = 6/group). An unpaired t-test was used for normally distributed data and the Mann-Whitney U test was used for non-normally distributed data. Data represent mean ± SEM. *P<0.05, **P<0.01, ***P<0.001, ****P<0.0001 vs SS group. SS, preoperative and anesthesia/surgery stress; SS+ABX, SS and antibiotics pretreatment before anesthesia/surgery stress.

To mechanistically determine the association between the anesthesia/surgery induced behavioral changes and gut microbiota, we evaluated the effects of treatment with antibiotics. We used an antibiotic cocktail containing ampicillin (0.1 mg/mL), streptomycin (0.5 mg/mL), and colistin (0.1 mg/mL) to ablate gut microbiota (17). Antibiotics treatment resulted in a marked elevation of the metabolites suberic acid (t[10]=4.05, *P=*0.002), 2-aminoadipic acid (Welch-corrected t[10.42]=6.669, *P<*0.001), N-methyl-L-glutamate (t[8]=5.791, *P<*0.001) and N-methyl-D-aspartic acid (Welch-corrected t[10.73]=3.065, *P=*0.002) in fecal samples of SS rats (**Figure 6A**) compared with those of untreated rats. In line with the alterations of metabolites, the antibiotics treatment in SS rats significantly minimized the emotional disorders (EPM, Welch-corrected t[17.68]=2.29, *P=*0.034, (**Figure 6B**); SPT, Welch-corrected t[19.13]=2.342, *P=*0.030, **Figure 6C**), reduced microglial activation (total dendritic branch length, t[22]=3.03, *P=*0.006; dendritic branch points, Welch-corrected t[18.5]=3.001, *P=*0.008; solidity, Welch-corrected t[19.37]=2.762, *P=*0.012; **Figure 6D**), and decreased pro-inflammatory cytokines (IL-6, Welch-corrected t[9.902]=2.444, *P=*0.035; TNF-ɑ, Welch-corrected t[9.087]=4.695, *P=*0.001; **Figure 6E**). Together, these results highlight the role of gut microbiota drove postoperative metabolite restructuring and behavioral deficits in rats with preoperative stress.

## Discussion

It was demonstrated that anesthesia/surgery significantly aggravates the anxiety- and depression-like behaviors, activates microglia, increases pro-inflammatory cytokines (IL-6 and TNF-α), induces microbiota dysbiosis, and promotes the restructuring of metabolites in rats suffering from preoperative stress. Furthermore, the treatment with probiotics before anesthesia/surgery could mitigate these detrimental effects, which strongly revealed that the abnormalities in the gut microbiota contribute to the development of psychiatric disorders in susceptible individuals. Finally, specific metabolites might be the key linkage between gut microbiota and postoperative cerebral dysfunction.

The clinical observation results revealed that the preoperative psychological stress (including anxiety and emergence depression) closely correlated with postoperative maladaptive behavioral changes (24, 25). In this present study, we focused on the contribution of adverse early-life experiences to aberrant brain maturation, which might underlie the basis for preoperative psychological stress. The adverse events *in utero* and during childhood increased the vulnerability to psychiatric diseases in individuals. Marion et al. examined the effects of multifactorial early-life adversities on behavior in mice exposed to a combination of maternal immune activation, maternal separation, and maternal unpredictable chronic mild stress. They found the offspring exposed to multi-hit early adversity showed sex-specific behavioral phenotypes, with males showing deficits in social behavior and females showing increased anxiety and compulsive behavior (26). In this study, it was found that rats exposed to a combination of prenatal stress and anesthesia/surgery stress presented exacerbated anxiety- and depression-like behaviors. It suggested that susceptible individuals were more prone to postoperative psychiatric disturbance. Similarly, Zhu et al. reported the changes in postoperative cognitive dysfunction in adult rats with the experience of maternal separation (27). Indeed, prenatal stress may not really compare with preoperative stress in humans, and further exploration of animal models is required in future studies.

As is revealed in accumulating evidence, the microbiota is one of the key regulators of the gut-brain axis. Besides, dysbiosis has been recognized to correlate with diseases in children and adults, including autism, attention deficit hyperactivity disorder, asthma, and allergies (28, 29). Compared with adults, the assembly of the microbiota in early life is susceptible to influences from environmental and experiential factors. Most importantly, these microbiota perturbations in the early-life can persist beyond infancy until childhood and adulthood (30–32). The influences of maternal stress during pregnancy on the offspring’s psychological function and behavior, and physical development and health are also linked to the microbiota (33, 34). Wu et al. identified that Enterococcus faecalis (E. faecalis), a bacterial species, could promote social activity and reduce corticosterone levels in mice after social stress (35). We also found the changes in gut microbiota profiles in rats with preoperative stress compared with the control rats. Interestingly, microbiota dysbiosis was exacerbated after anesthesia/surgery exposure.

The association between anesthesia/surgery and gut microbiota has been confirmed by the cohorts of various sizes. Zhang et al. proposed that there were different gut microbiota compositions between the 8 weeks old mice with postoperative delirium-like behaviors and the control mice. Specifically, the former had a higher level of gammaproteobacterial, whereas the latter had higher levels of tenericutes and mollicutes (36). Liu et al. found that anesthesia/surgery caused reduction in the levels of gut lactobacillus between the 18 and 9 months old mice (12). Consistently, the data from the present studies demonstrated that the rats with exacerbated postoperative anxiety- and depression-like behaviors had a lower level of Lactobacillus. It was well documented that the use of antibiotics results in both the short-term and long-term alterations in the composition of the gut microbiome (37–39). Luo et al. proved that pretreating mice with compound antibiotics could prevent the learning and memory deficits induced by anesthesia/surgery (40). Here, the exacerbated psychological disturbances and neuro-inflammation in rats with preoperative stress were also alleviated when pretreated with antibiotics. However, the current results could not prove whether the changes in pro-inflammatory cytokines are metabolite-driven or the effects of antibiotics. Future treatment should also be designed to systematically determine the interaction between anesthesia/surgery and the treatment with antibiotics on the changes of gut microbiota in rats.

Accumulated evidence has demonstrated that the metabolites of the gut microbiota could enter the circulatory system and regulate the physiologies and behaviors of hosts (16, 41–43). Significant correlations between metabolites and microbiota at genus and species levels were found, suggesting that postoperative metabolic restructuring in rats with preoperative stress might be derived from gut microbiota. Previous studies have suggested the direct or indirect involvement of bacterial metabolites, and particularly, short-chain fatty acids and bile acids in the development of some neurological disorders (44–46). As revealed by the non-targeted metabolomics technique in our current studies, the most significant metabolic changes after anesthesia/surgery in rats exposed to preoperative stress were amino acid metabolomics and organic acid derivatives. Notably, 2-aminoadipic acid and suberic acid are associated with depression and autism-related behaviors (47, 48). It has been reported that other amino acids metabolomics, such as N-methyl-D-aspartic acid, influence multiple neuropsychiatric disorders including anxiety, depression, and schizophrenia (49, 50). Here, it was confirmed that 2-aminoadipic acid and N-methyl-L-glutamate were associated with the behavioral performance in the EPM and SPT, and the treatment with antibiotics normalized the metabolite levels and prevented the behavioral deficits. The results from the current studies highlighted the role of gut microbiota that could drive the metabolite restructuring in aggravated behavioral deficits after anesthesia/surgery in rats with preoperative stress. The metabolites (eg, 2-aminoadipic acid, N-methyl-L-glutamate) could be used as early diagnostic biomarkers for susceptibility subjects to postoperative cerebral dysfunction. Future studies should be carried out to identify the key bacteria strains that account for the metabolites changes with the current system. On the other hand, further studies should be performed to determine the underlying mechanism and the clinical relevance of specific metabolites in psychological deficits and neuroinflammation should also be warranted.

In conclusion, the present study sheds light on the relationship among metabolites, gut microbiota, and neuroinflammation after anesthesia/surgery in rats with preoperative stress. The results proved that abnormalities in the gut microbiota contribute to postoperative metabolic restructuring, neuroinflammation, and psychiatric deficits in susceptible individuals. Future therapeutic interventions could be developed for postoperative psychiatric disorders by targeting specific microbiota-related metabolites.

## Data availability statement

The datasets presented in this study can be found in online repositories. The names of the repository/repositories and accession number(s) can be found below: BioProject database, accession number PRJNA782482.

## Ethics statement

The animal study was reviewed and approved by Animal Care and Use Committee of The First Affiliated Hospital of Zhengzhou University.

## Author contributions

J-JY designed the study. LL, MJ, HG, and JY performed the experiments. LL and SC analyzed the data. LL and CS analyzed the data. LL drafted the original manuscript. All authors contributed to the article and approved the submitted version.

## Funding

This work was supported by the grant from the National Natural Science Foundation of China (No. 82001157).

## Conflict of interest

The authors declare that the research was conducted in the absence of any commercial or financial relationships that could be construed as a potential conflict of interest.

## Publisher’s note

All claims expressed in this article are solely those of the authors and do not necessarily represent those of their affiliated organizations, or those of the publisher, the editors and the reviewers. Any product that may be evaluated in this article, or claim that may be made by its manufacturer, is not guaranteed or endorsed by the publisher.

## References

[1] McBride KE Solomon MJ Bannon PG Glozier N Steffens D. Surgical outcomes for people with serious mental illness are poorer than for other patients: a systematic review and meta-analysis. Med J Aust (2021) 214(8):379–85. doi: 10.5694/mja2.51009 33847005

[2] Orri M Boleslawski E Regimbeau JM Barry C Hassler C Gregoire E. Influence of depression on recovery after major noncardiac surgery: a prospective cohort study. Ann Surg (2015) 262(8):882–9. doi: 10.1097/SLA.0000000000001448 26583680

[3] Chaichana KL Mukherjee D Adogwa O Cheng JS McGirt MJ. Correlation of preoperative depression and somatic perception scales with postoperative disability and quality of life after lumbar discectomy. J Neurosurg Spine (2011) 14(2):261–7. doi: 10.3171/2010.10.SPINE10190 21214315

[4] Umholtz M Cilnyk J Wang CK Porhomayon J Pourafkari L Nader ND. Postanesthesia emergence in patients with post-traumatic stress disorder. J Clin Anesth (2016) 34:3–10. doi: 10.1016/j.jclinane.2016.02.047 27687337

[5] Short AK Baram TZ. Early-life adversity and neurological disease: age-old questions and novel answers. Nat Rev Neurol (2019) 15(11):657–69. doi: 10.1038/s41582-019-0246-5 PMC7261498 31530940

[6] Klengel T Binder EB. Epigenetics of stress-related psychiatric disorders and Gene×Environment interactions. Neuron (2015) 86(6):1343–57. doi: 10.1016/j.neuron.2015.05.036 26087162

[7] Caspi A Sugden K Moffitt TE Taylor A Craig IW Harrington H. Influence of life stress on depression: moderation by a polymorphism in the 5-HTT gene. Science (2003) 301(5631):386–9. doi: 10.1126/science.1083968 12869766

[8] Haapanen MJ Perälä MM Salonen MK Kajantie E Simonen M Pohjolainen P. Early life stress and frailty in old age: the Helsinki birth cohort study. BMC Geriatr (2018) 18(1):179. doi: 10.1186/s12877-018-0873-5 30103697 PMC6090686

[9] Rod NH Bengtsson J Budtz-Jørgensen E Clipet-Jensen C Taylor-Robinson D Andersen MN. Trajectories of childhood adversity and mortality in early adulthood: a population-based cohort study. Lancet (2020) 396(10249):489–97. doi: 10.1016/S0140-6736(20)30621-8 32798491

[10] Bolton JL Molet J Regev L Chen Y Rismanchi N Haddad E. Anhedonia following early-life adversity involves aberrant interaction of reward and anxiety circuits and is reversed by partial silencing of amygdala corticotropin-releasing hormone gene. Biol Psychiatry (2018) 83(2):137–47. doi: 10.1016/j.biopsych.2017.08.023 PMC5723546 29033027

[11] Deiner S Liu X Lin HM Jacoby R Kim J Baxter MG. Does postoperative cognitive decline result in new disability after surgery? Ann Surg (2020) 274:e1108–14. doi: 10.1097/SLA.0000000000003764 32149824

[12] Liu FN Liu L Shen S Jiang Z Dong Y Wang Y. Anesthesia and surgery induce age-dependent changes in behaviors and microbiota. Aging (Albany NY) (2015) 12(2):1965–86. doi: 10.18632/aging.102736 PMC7053599 31974315

[13] Needham BD Kaddurah-Daouk R Mazmanian SK. Gut microbial molecules in behavioural and neurodegenerative conditions. Nat Rev Neurosci (2020) 21(12):717–31. doi: 10.1038/s41583-020-00381-0 33067567

[14] Liu Z Dai X Zhang H Shi R Hui Y Jin X. Gut microbiota mediates intermittent-fasting alleviation of diabetes-induced cognitive impairment. Nat Commun (2020) 11(1):855. doi: 10.1038/s41467-020-14676-4 32071312 PMC7029019

[15] Bauer KC Huus KE Finlay BB. Microbes and the mind: emerging hallmarks of the gut microbiota-brain axis. Cell Microbiol (2016) 18:632–44. doi: 10.1111/cmi.12585 26918908

[16] Rogers GB Keating DJ Young RL Wong ML Licinio J Wesselingh S. From gut dysbiosis to altered brain function and mental illness: mechanisms and pathways. Mol Psychiatry (2016) 21:738–48. doi: 10.1038/mp.2016.50 PMC4879184 27090305

[17] Vicentini FA Mathews AJ Pittman QJ Swain MG Sharkey KA Hirota SA. Behavioural adaptations after antibiotic treatment in male mice are reversed by activation of the aryl hydrocarbon receptor. Brain Behav Immun (2021) 98:317–29. doi: 10.1016/j.bbi.2021.08.228 34461234

[18] Marrocco J Reynaert ML Gatta E Gabriel C Mocaër E Di PS. The effects of antidepressant treatment in prenatally stressed rats support the glutamatergic hypothesis of stress-related disorders. J Neurosci (2014) 34(6):2015–24. doi: 10.1523/JNEUROSCI.4131-13.2014 PMC6608531 24501344

[19] Qiu LL Ji MH Zhang H Yang JJ Sun XR Tang H. NADPH oxidase 2-derived reactive oxygen species in the hippocampus might contribute to microglial activation in postoperative cognitive dysfunction in aged mice. Brain Behav Immun (2016) 51:109–18. doi: 10.1016/j.bbi.2015.08.002 26254234

[20] Ji MH Lei L Gao DP Tong JH Wang Y Yang JJ. Neural network disturbance in the medial prefrontal cortex might contribute to cognitive impairments induced by neuroinflammation. Brain Behav Immun (2020) 89:133–44. doi: 10.1016/j.bbi.2020.06.001 32505714

[21] Liu Q Sun YM Huang H Chen C Wan J Ma LH. Sirtuin 3 protects against anesthesia/surgery-induced cognitive decline in aged mice by suppressing hippocampal neuroinflammation. J Neuroinflamm (2021) 18(1):41. doi: 10.1186/s12974-021-02089-z PMC7863360 33541361

[22] Wang Z Chen WH Li SX He ZM Zhu WL Ji YB. Gut microbiota modulates the inflammatory response and cognitive impairment induced by sleep deprivation. Mol Psychiatry (2021) 26(11):6277–92. doi: 10.1038/s41380-021-01113-1 33963281

[23] Schmidtner AK Slattery DA Gläsner J Hiergeist A Gryksa K Malik VA. Minocycline alters behavior, microglia and the gut microbiome in a trait-anxiety-dependent manner. Transl Psychiatry (2019) 9(1):223. doi: 10.1038/s41398-019-0556-9 31519869 PMC6744405

[24] Kain ZN Mayes LC Caldwell-Andrews AA Karas DE McClain BC. Preoperative anxiety, postoperative pain, and behavioral recovery in young children undergoing surgery. Pediatrics (2006) 118(2):651–8. doi: 10.1542/peds.2005-2920 16882820

[25] Reddy SK Deutsch N. Behavioral and emotional disorders in children and their anesthetic implications. Children (Basel) (2020) 7(12):253. doi: 10.3390/children7120253 PMC7759846 33255535

[26] Marion R Philippe A Julien C Pierre-Antoine G Lilian B de Oliveira M. Multi-hit early life adversity affects gut microbiota, brain and behavior in a sex-dependent manner. Brain Behav Immun (2019) 80:179–92. doi: 10.1016/j.bbi.2019.03.006 30872090

[27] Zhu Y Wang Y Yao R Hao T Cao J Huang H. Enhanced neuroinflammation mediated by DNA methylation of the glucocorticoid receptor triggers cognitive dysfunction after sevoflurane anesthesia in adult rats subjected to maternal separation during the neonatal period. J Neuroinflamm (2017) 14(1):6. doi: 10.1186/s12974-016-0782-5 PMC5234142 28086911

[28] Codagnone MG Spichak S O’Mahony SM O’Leary OF Clarke G Stanton C. Programming bugs: microbiota and the developmental origins of brain health and disease. Biol Psychiatry (2019) 85(2):150–63. doi: 10.1016/j.biopsych.2018.06.014 30064690

[29] Bloemendaal M Szopinska-Tokov J Belzer C Boverhoff D Papalini S Michels F. Probiotics-induced changes in gut microbial composition and its effects on cognitive performance after stress: exploratory analyses. Transl Psychiatry (2021) 11(1):300. doi: 10.1038/s41398-021-01404-9 34016947 PMC8137885

[30] Streit F Prandovszky E Send T Zillich L Frank J Sabunciyan S. Microbiome profiles are associated with cognitive functioning in 45-month-old children. Brain Behav Immun (2021) 98:151–60. doi: 10.1016/j.bbi.2021.08.001 34371134

[31] De MP Henriques AC Azevedo R Sá SI Cardoso A Fonseca B. Gut microbiome composition and metabolic status are differently affected by early exposure to unhealthy diets in a rat model. Nutrients (2021) 13(9):3236. doi: 10.3390/nu13093236 34579113 PMC8469890

[32] Ronan V Yeasin R Claud EC. Childhood development and the microbiome-the intestinal microbiota in maintenance of health and development of disease during childhood development. Gastroenterology (2021) 160(2):495–506. doi: 10.1053/j.gastro.2020.08.065 33307032 PMC8714606

[33] De PG Blennerhassett P Lu J Deng Y Park AJ Green W. Microbiota and host determinants of behavioural phenotype in maternally separated mice. Nat Commun (2015) 6:7735. doi: 10.1038/ncomms8735 26218677

[34] Dawson SL O’Hely M Jacka FN Ponsonby AL Symeonides C Loughman A. Maternal prenatal gut microbiota composition predicts child behaviour. EBioMedicine (2021) 68:103400. doi: 10.1016/j.ebiom.2021.103400 34098340 PMC8190443

[35] Wu W-L Adame MD Liou C-W Barlow JT Lai T-T Sharon G. Microbiota regulate social behaviour *via* stress response neurons in the brain. Nature (2021) 595(7867):409–14. doi: 10.1038/s41586-021-03669-y PMC8346519 34194038

[36] Zhang J Bi JJ Guo GJ Yang L Zhu B Zhan GF. Abnormal composition of gut microbiota contributes to delirium-like behaviors after abdominal surgery in mice. CNS Neurosci Ther (2019) 25(6):685–96. doi: 10.1111/cns.13103 PMC6515708 30680947

[37] Bercik P Denou E Collins J Jackson W Lu J Jury J. The intestinal microbiota affect central levels of brain-derived neurotropic factor and behavior in mice. Gastroenterology (2011) 141:599–609.e3. doi: 10.1053/j.gastro.2011.04.052 21683077

[38] Desbonnet L Clarke G Traplin A O’Sullivan O Crispie F Moloney RD. Gut microbiota depletion from early adolescence in mice: implications for brain and behaviour. Brain Behav Immun (2015) 48:165–73. doi: 10.1016/j.bbi.2015.04.004. 25866195

[39] Sampson TR Debelius JW Thron T Janssen S Shastri GG Ilhan ZE. Gut microbiota regulate motor deficits and neuroinflammation in a model of parkinson’s disease. Cell (2016) 167:1469–80.e12. doi: 10.1016/j.cell.2016.11.018 27912057 PMC5718049

[40] Luo AL Li S Wang X Xie Z Li SY Hua DY. Cefazolin improves anesthesia and surgery-induced cognitive impairments by modulating blood-brain barrier function, gut bacteria and short chain fatty acids. Front Aging Neurosci (2021) 13:748637. doi: 10.3389/fnagi.2021.748637 34720997 PMC8548472

[41] Han S Van TW Fischer CR Merrill BD DeFelice BC Sanchez JM. A metabolomics pipeline for the mechanistic interrogation of the gut microbiome. Nature (2021) 595(7867):415–20. doi: 10.1038/s41586-021-03707-9 PMC8939302 34262212

[42] Matsuda Y Ozawa N Shinozaki T Wakabayashi K-I Suzuki K Kawano Y. Ergothioneine, a metabolite of the gut bacterium lactobacillus reuteri, protects against stress-induced sleep disturbances. Transl Psychiatry (2020) 10(1):170. doi: 10.1038/s41398-020-0855-1 32467627 PMC7256047

[43] Han H Yi B Zhong RQ Wang MY Zhang SF Ma J. From gut microbiota to host appetite: gut microbiota-derived metabolites as key regulators. Microbiome (2021) 9(1):162. doi: 10.1186/s40168-021-01093-y 34284827 PMC8293578

[44] Grant SM DeMorrow S. Bile acid signaling in neurodegenerative and neurological disorders. Int J Mol Sci (2020) 21(17):5982. doi: 10.3390/ijms21175982 PMC7503576 32825239

[45] Tian PJ Zhu HY Qian X Chen Y Wang Z Zhao JX. Consumption of butylated starch alleviates the chronic restraint stress-induced neurobehavioral and gut barrier deficits through reshaping the gut microbiota. Front Immunol (2021) 12:755481. doi: 10.3389/fimmu.2021.755481 34603341 PMC8485752

[46] Wu M Tian T Mao Q Zou T Zhou C-J Xie J. Associations between disordered gut microbiota and changes of neurotransmitters and short-chain fatty acids in depressed mice. Transl Psychiatry (2020) 10(1):350. doi: 10.1038/s41398-020-01038-3 33067412 PMC7567879

[47] Puig-Alcaraz C Fuentes-Albero M Cauli O. Relationship between adipic acid concentration and the core symptoms of autism spectrum disorders. Psychiatry Res (2016) 242:39–45. doi: 10.1016/j.psychres.2016.05.027 27259135

[48] David J Gormley S McIntosh AL Kebede V Thuery G Varidaki A. L-alpha-amino adipic acid provokes depression-like behaviour and a stress related increase in dendritic spine density in the pre-limbic cortex and hippocampus in rodents. Behav Brain Res (2019) 362:90–102. doi: 10.1016/j.bbr.2019.01.015 30639510

[49] Deutschenbaur L Beck J Kiyhankhadiv A Mühlhauser M Borgwardt S Walter M. Role of calcium, glutamate and NMDA in major depression and therapeutic application. Prog Neuropsychopharmacol Biol Psychiatry (2016) 64:325–33. doi: 10.1016/j.pnpbp.2015.02.015 25747801

[50] McCutcheon RA Krystal JH Howes OD. Dopamine and glutamate in schizophrenia: biology, symptoms and treatment. World Psychiatry (2020) 19(1):15–33. doi: 10.1002/wps.20693 31922684 PMC6953551

